# Prevalence and contributors to anaemia among children aged 6 to 59 months in Kyangwali Refugee settlement, Western Uganda: a cross-sectional study

**DOI:** 10.1186/s12887-024-05377-8

**Published:** 2025-01-11

**Authors:** Christine Nakimera, Philip Bright Bwajojo, William Kaweesa, Joan Nampiima, Faith Chebet, Sharifah Namuwawu, Martin Bwemage, Moses Nuwabasa, Regina Ndagire, Allan Lugaajju, Joel Tumwebaze, Catherine Nassozi Lwanira

**Affiliations:** 1 Institute of Public Health and Management, Clarke International University, Kampala, P.O. Box 7782, Uganda; 2 Community Integrated Development Initiatives, Kyangwali Refugee settlement, Kampala, P.O. Box 692, Uganda; 3 Action Against Hunger, Kampala, P.O. Box 3177, Uganda; 4 Medical Teams International, Kampala, P.O. Box 26073, Uganda; 5 Strong Minds, Kampala, P.O. Box 35874, Uganda; 6 Department of Research, School of Graduate studies, Research and Innovations, Clarke International University, Kampala, P.O. Box 7782, Uganda; 7 School of Biomedical Sciences, College of Health Sciences, Makerere University, Kampala, P.O. Box 7072, Uganda

**Keywords:** Anaemia, Refugee settlement, Uganda

## Abstract

**Background:**

Anaemia is a major cause of morbidity among children under five years in Uganda. However, its magnitude among refugee populations is marginally documented. In this study, the prevalence and contributors to anaemia among children 6 to 59 months in Kyangwali refugee settlement in Western Uganda was determined.

**Methods:**

This was a cross-sectional study that was carried out among 415 mother- child dyads at Kwangwali refugee settlement. Anaemia was determined by measuring haemoglobin concentration using the HaemoCue method, while nutritional status was examined using standard World Health Organisation (WHO) nutritional indices. Data abstraction forms, pretested questionnaires and face to face interviews were used to collect patient data. Associations between the independent variables and anaemia were examined using modified Poisson regression with robust standard errors. In all statistical tests, a P- value of < 0.05 was considered as significant.

**Results:**

The proportion of children with anaemia was 49.2% (95% CI: 44.4–53.9). Anaemia was 1.4 times (95% CI = 1.13–1.82; *p* = 0.003) more prevalent among wasted children than the normal children. The prevalence of anaemia was also higher among underweight children than those with normal weight (aPR = 1.37, 95% CI = 1.11–1.70; *p* = 0.004). Additionally, the prevalence of anaemia was higher among children of birth order of 6 or above (aPR = 2.00, 95% CI = 1.22–3.29; *p* = 0.006), while anaemia prevalence was lower among children whose mothers’ had attained secondary level of education (aPR = 0.19, CI = 0.04–0.98; *p* = 0.048) and those who fed on fish (aPR = 0.75, 95% CI = 0.57–0.99; *p* = 0.039) and meals prepared with oils and fats (aPR = 0.70, 95% CI = 0.51–0.97; *p* = 0.029). There was no significant relationship between anaemia occurrence and the household dietary diversity score.

**Conclusions:**

About half of the study children were found to be anaemic. The most significant contributors to anaemia in the study population were malnutrition, maternal education, feeding practices and birth order. The study findings suggest need of screening of children for anaemia at all nutritional clinics, promotion of education, addressing barriers to sustainable food supply and accessibility of nutrient-dense foods, treating anaemia in children alongside other micronutrient deficiencies and addressing the nutritional needs of multiparous mothers in refugee communities.

## Background

Anaemia remains a major cause of morbidity among children and women [1–3]. Globally, more than 40% of children under five years are afflicted by anaemia [2, 3]. The greatest burden of childhood anaemia is reported in the African region, where the overall prevalence reaches up to 60% [1, 4, 5]. Uganda has one of the highest prevalence rates of anaemia among children 6–59 months, estimated at 52% in 2019 [6]. This proportion has not changed significantly from the earlier estimate of 53% in 2016 [6, 7]. Refugee populations are particularly at risk of developing anaemia due to food insecurity, overcrowding, inadequate sanitation and high morbidity mainly due to malaria and diarrhoea [8, 9] with prevalence rates reaching 20% and 15% respectively among children living in refugee communities of Uganda [9]. Globally, anaemia affects over 44% of children living in refugee settlements [8], while the prevalence of childhood anaemia reaches up to 50% in some refugee communities of Africa [10]. Uganda hosts over 1.5 million refugees and asylum seekers in thirteen refugee settlements [9, 11]. The prevalence of anaemia in all of the thirteen Ugandan refugee settlements exceeds the acceptable threshold of less than 20% [9], with the highest burden currently reported in Kyangwali, Palabek, Kiryandongo and Imvepi refugee settlements [9]. Anaemia can have severe effects in children including stunted growth, impaired cognitive and motor development and increased susceptibility to infections [1, 12]. Additionally, children admitted with anaemia and malaria parasitaemia may have increased likelihood of recurrent severe anaemia [13] and high risk of post-discharge mortality [13, 14].

Anaemia in children has been associated with several factors including age [3, 5, 15–18], sex [3–5, 16, 17, 19–21], birth order [17, 21], recent illnesses (such as fever [5, 17, 20, 21], malaria [16, 18], diarrhoea [17, 19, 21] and acute respiratory infections [17]), meal frequency [19], vegetable and fruit consumption [19], malnutrition or stunting [15, 17, 19, 21, 22], deworming [21], maternal age [5, 17, 21], education [4, 15, 17, 20, 21], anaemia status [5, 16–18, 21] and body mass index [5], current maternal pregnancy [5], multiple births [21], maternal health [5], family structure [5], family size [4, 5, 15], place of residence [4, 15, 17, 18], geographical setting [3], water/sanitation [5] and wealth index [5, 17, 18, 20, 21]. However, many of these studies have primarily focused on non-refugee populations. There is still a paucity of information regarding the burden and determinants of anaemia among children under five years living in displaced communities. A recent study conducted among children aged 6 months to 15 years at Debark refugee camp, Northwest Ethiopia found anaemia to be associated with dietary diversity, presence of diarrhoea and fever, wasting and duration of stay in the camp for more than six months [23].

Over the years, the government of Uganda through its implementing partners adopted a multisectoral approach to reduce anaemia including programs for maternal anemia, integrated deworming, iron–folic acid (IFA) supplementation into facility-based antenatal care [24, 25] and malaria prevention [26]. Through aid provided by the United Nations High Commission for Refugees (UNHCR) and the United Nations World Food Programme (WFP), there has been effort to provide refugee communities free access to health services and nutritional support to pregnant/lactating mothers and infants [27, 28]. Refugee communities are also provided with some General Food Assistance (GFA) towards food and other livelihood needs [28, 29]. Vitamin A supplementation, measles vaccination and deworming practices are generally adequate across all refugee settlements and host districts, except in Kiryandongo settlement, Kiryandongo district and Kikuube district where deworming is at 57.8, 54.5 and 58.2% [9]. Despite these efforts, anaemia remains persistently high in refugee populations. Moreover, the lack of sufficient information on the burden and determinants of anaemia in these communities impedes potential strategies for reducing anaemia in highly burdened refugee communities such as Kyangwali refugee settlement. In this study, the prevalence and contributors to anaemia among children 6 to 59 months in Kyangwali refugee settlement in Western Uganda was determined.

## Methods

### Study design and setting

**Fig. 1:**
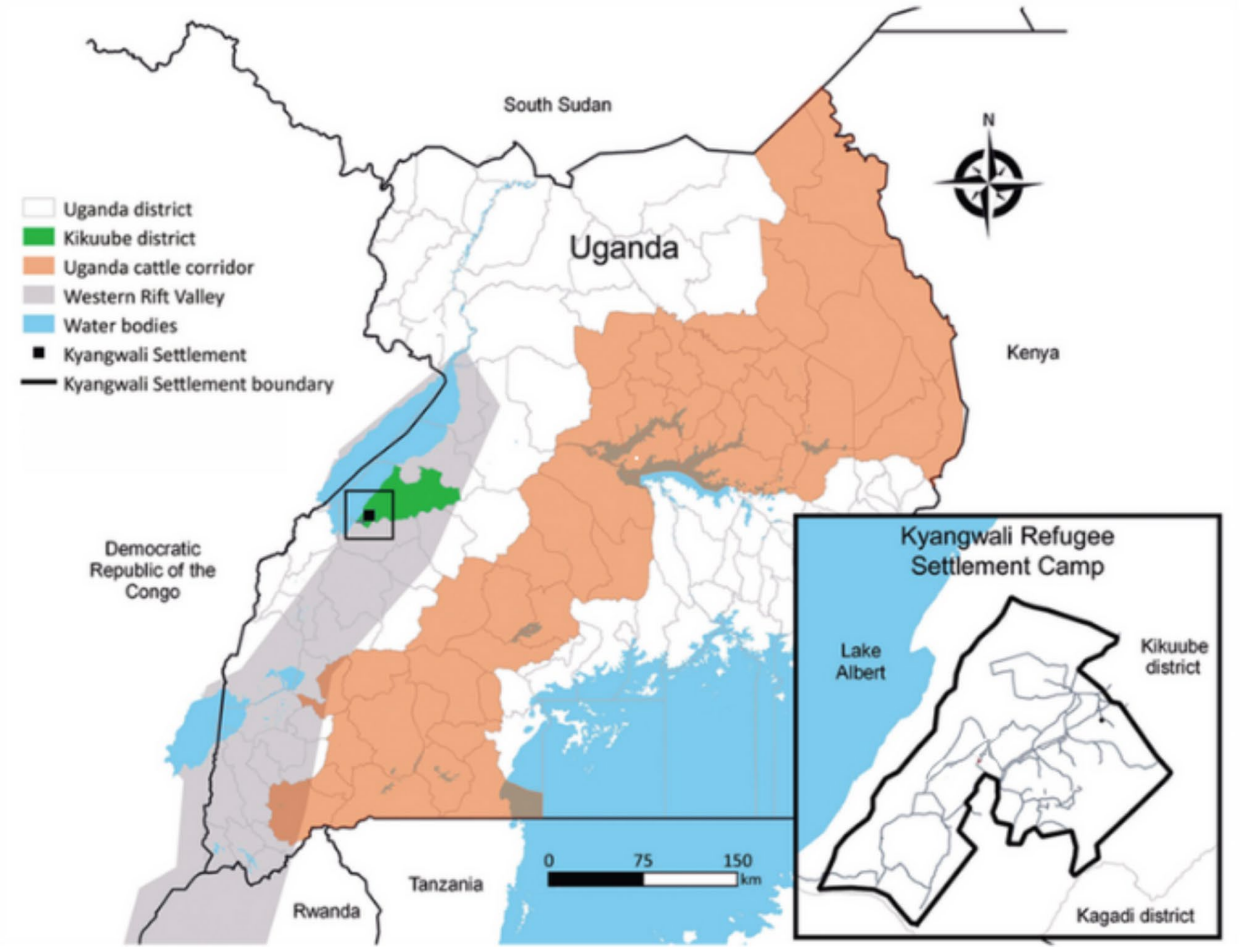
Map of Uganda showing the location of Kyangwali Refugee settlement (black box); Adapted from Nyakarahuka et al. [49],

This was a cross-sectional study that was carried out between November, 2022 to April, 2023 in Kyangwali refugee settlement. Kyangwali refugee settlement is one of the 13 refugee settlements in Uganda [11]. It is located in Kyangwali sub-county, Kikuube district (formerly part of Hoima district) in western Uganda (Fig. 1**).** This refugee settlement comprises 13 zones with an estimated population of 125,786 refugees, which is approximately 28.2% of the total projected population of Kikuube district (446,578 people). Majority of the residents are immigrants from the Democratic Republic of Congo (96.7%) and South Sudan (2.7%), while the rest are from Rwanda, Burundi, Kenya and Somalia. The greater population are women and children (83%), with children under five accounting for about 20% of the refugee population [30, 31]. Less than half of the refugees (42%) have an occupation, mainly employed as farmers [30]. Health services within the refugee settlement are provided through government and UNHCR-supported partner-run health facilities comprising one health centre (HC) IV, two HC IIIs and seven HC IIs [30].

### Study population

The study population included mother - child dyads who received care at the various health facilities within Kyangwali refugee settlement. The child was included in the study if they (a) resided in Kyangwali refugee settlement (b) were of age between 6 and 59 months and (c) if the mother provided written informed consent to participate in the study. Children who required emergency treatment were excluded from the study.

### Sample size estimation

The study sample size was estimated using Kish and Leslie formula of sample size estimation [32] using a Zα score corresponding to 95% confidence interval (Zα) of 1.96, an estimated prevalence of anaemia among children under five in refugee settlements (p) of 53% [29], a level of precision or maximum acceptable error (d) of 5%. After correcting for a 10% non-response, the study sample size was 421.

### Sampling procedure

Participants were enrolled from five randomly selected health facilities that included Maratatu B HC III, Maratatu D HC II, Rwenyawawa HC III, Kasonga HC II and Kavule HC II. Each of the health facilities provides young child clinic (YCC) services once a week to an estimated 40–115 children, who formed the accessible population for this study. Participants were recruited on the YCC day by systematic random sampling using the estimated accessible population as the sampling frame. Every fourth (4th ) eligible participant was enrolled into the study. The sample size was evenly distributed among the five selected facilities and on average, a total of 85 child-mother pairs were taken up from each facility.

### Data collection methods

Patient examination was done by the attending physicians at the health facilities as part of the routine health care. Participants’ data including the child and mother’s demographics, comorbidities and medical history was extracted from the patient files using the data abstraction forms. A structured questionnaire was used to collect the other data on house-hold and nutritional-related factors. The data abstraction tool and the questionnaire were developed following findings and tools used in previous similar studies [15]. The tools were pretested at Kyangwali HC IV and thereafter adjusted to meet the study population and objectives.

### Measurement of study variables

#### Haemoglobin level determination

Haemoglobin (Hb) concentration was assayed for both study children and the mothers using the HaemoCue method as described elsewhere [33, 34]. This was done at the outpatient departments of the different health facilities by trained laboratory personnel. Briefly, blood was obtained from each child and mother by the heel or finger prick and collected into a disposable cuvette. Haemoglobin measurement was done using the HaemoCue Hb 301 analyzer and the concentration was recorded to the nearest 0.1 g per deciliter (g/dL). The accuracy of the HaemoCue was ensured by using a controlled cuvette daily [33]. The Hb level was then classified into severe anaemia (Hb < 7.0 g/dL), moderate anaemia (Hb = 7.0–9.9 g/dL), mild anaemia (Hb = 10–10.9 g/dL) and not anaemic (Hb ≥ 11 g/dL) [35].

#### Nutritional status assessment

The children were assessed for nutritional status using standard anthropometric measurements [36]. Each child’s weight was measured using a Seca weighing scale, which was calibrated to zero. If the child could not stand on the weighing scale, both mother and child were weighed together. Then, the weight of the child was obtained by subtracting the mother’s weight from the measurement of both the child and mother. The weight was determined in triplicate and recorded to the nearest first decimal place in kilograms (kg). To measure the length or height, the stadiometer was placed either horizontally or vertically on a flat ground surface and the child’s length or height was recorded. Children who were less than 2 years were measured while lying down in an appropriate position as their mothers held them. The height of the children was determined in triplicate and recorded to the nearest first decimal place in cm. The WHO nutritional indices based on WHO 2006 child growth standards were calculated using ENA software version 2022. Nutritional status was reported as underweight, stunting and acute malnutrition or wasting. Underweight was defined as weight-for-age Z-score (WAZ) <-2 standard deviation (SD) of the median, stunting as height-for-age Z-score (HAZ) <-2 SD of the median and wasting as weight-for-height Z-score (WHZ) <-2 SD of the median [36].

#### Household dietary diversity measurement

Household dietary diversity (HDD) was determined using a questionnaire on the food varieties consumed in the household. The tool was informed by the recommended food groups [37] which include cereals; white roots and tubers; vitamin A rich vegetables and tubers; dark green leafy vegetables; other vegetables; vitamin A rich fruits; other fruits; organ meat; flesh meat; eggs; fish and seafood; legumes, nuts and seeds; milk and products; oils and fats; sweets; and spices, condiments and beverages. Participants were asked to describe the foods or drinks that they had in the last 24 h. The responses were captured as yes = 1 or no = 0 against each food item from which an HDD score was computed for each participant by aggregating the score on each food item. The highest possible HDD score was 12.

#### Data management and analysis

Data was entered and cleaned using EpiData version 4.6.0.2. The dataset was then exported to STATA 16.0 statistical software (StataCorp, College Station, Texas, USA) where analysis was done. Means and standard deviations (SD) were used to summarize normally distributed continuous data, while medians and interquartile ranges (IQR) were used for skewed continuous data. For categorical variables, proportions and frequencies were used. For analysis, the outcome was dichotomized into anaemia (Hb levels < 11 g/dl) and no anaemia (Hb levels ≥ 11 g/dl). The prevalence of anaemia was determined by dividing the number of children who had Hb levels < 11 g/dl by the study sample size.

The association between the independent variables and anaemia was examined using modified Poisson regression model with robust standard errors. Prevalence ratios (PRs) were calculated considering a 5% significance level and 95% level of confidence. All statistical tests were two-tailed. Variables with a p-value < 0.2 at bivariate were considered for multivariable analysis. The chunk test was used to assess for interaction by comparing the reduced model (without interaction terms) with the full model (with interaction terms) using the likelihood ratios [38], while confounding was assessed using a 10% change in crude and adjusted PR. There was no interaction or confounding. Variables with a p-value < 0.05 after multivariable analysis were considered to be the factors that are associated with anaemia in the study population.

## Results

### Baseline characteristics of the study population

The study enrolled 421 mother-child dyads but only 415 were considered in the final analysis. Six questionnaires were excluded for being incomplete. Of all child participants, 234 (56.4%) were female. The median (IQR) age of the children was 42.7(34.8, 47.1) months with 223 (53.7%) over 42 months of age. More than two-thirds of the children (75.4%) were stunted and 32.8% had malaria. Slightly over half of the mothers (54%) were less than 25 years of age and 233 (56.1%) had no formal education (Table 1).

**Table 1:** Baseline characteristics of the children and the mothers (*N* = 415)

Variables	Categories	Frequency (*n*)	Percentage (%)
Sex of child	Male	181	43.6
	Female	234	56.4
Child’s age (in months)	< 24.9	86	20.7
	25-41.9	106	25.5
	≥ 42	223	53.7
Weight (kgs), mean (SD)	11.7 ± 2.5		
Birth weight (kgs)	< 2.5	51	12.3
	≥ 2.5	364	87.7
Birth order	1–3	314	75.7
	4–5	88	21.2
	6 and above	13	3.1
Child’s nutritional status			
	Wasted (WHZ <-2 SD)	31^a^	7.5
	Underweight (WAZ <-2 SD)	178^a^	42.9
	Stunted (HAZ <-2 SD)	313^a^	75.4
Comorbidities in child	None	196	47.2
	Fever	34	8.2
	Malaria	136	32.8
	Diarrhoea	34	8.2
	Acute respiratory infections	15	3.6
Mother’s age (years)	< 25	224	54.0
	26–35	169	40.7
	Above 36	22	5.3
Mother’s Education level	No formal education	233	56.1
	Primary	171	41.2
	Secondary	11	2.7
Number of children	1–2	174	41.9
	3–5	164	39.5
	> 5	77	18.6

SD- Standard deviation; WHZ- Weight-for-Height Z score; WAZ-Weight -for-age Z score; HAZ- Height-for-age Z score

^a^ Indicator only shows the number of children under each category; who were stunted, underweight and stunted out of the total study population

### Prevalence of anemia among children 6 to 59 months in Kyangwali Refugee settlement

All study children were assessed for their anemia status using standard haemoglobin measurements. The median (IQR) haemoglobin value was 11 (9,11.5) g/dL {reference normal range, ≥ 11 g/dL} [35]. Half of the children (50.8%) had normal haemoglobin levels (≥ 11 g/dL). The proportion of children with mild anaemia (Hb = 10–10.9 g/dL) was 18.8%, 100 of 415 children (24.1%) had moderate anaemia (Hb = 7.0–9.9 g/dL) and 6.3% (26 of 415 children) had severe anaemia (Hb < 7.0 g/dL). Overall, the proportion of children with anaemia was 49.2% (95% CI: 44.4–53.9).

### Factors associated with child anaemia in Kyangwali Refugee settlement

This study determined the factors associated with the occurrence of anaemia among children 6 to 59 months. Anaemia prevalence was found to be independently associated with a child’s nutritional status, birth order and diet. According to the study findings, anaemia was 1.4 times (95% CI = 1.13–1.82; *p* = 0.003) more prevalent among wasted children than the normal children. The prevalence of anaemia was also higher among underweight children than those with normal weight (aPR = 1.37, 95% CI = 1.11–1.70; *p* = 0.004).

Other factors that significantly affected anaemia occurrence were being birth order of 6 or above (aPR = 2.00, CI = 1.22–3.29; *p* = 0.006), mother having secondary level of education (aPR = 0.19, CI = 0.04–0.98; *p* = 0.048) and type of food, with less anaemia prevalence reported among children who fed on fish (aPR = 0.75, CI = 0.57–0.99; *p* = 0.039) and meals prepared with oils and fats (aPR = 0.70, CI = 0.51–0.97; *p* = 0.029). However, there was no significant relationship between anaemia occurrence and the household dietary diversity score. Details of the bivariate and multivariate analyses of the determinants of anaemia prevalence among the children are given in Table 2.

**Table 2:** Bivariate and multivariable analysis of the determinants of anaemia prevalence among children 6–59 months

Characteristic	Anaemic		cPR	95%CI	*p*-value	aPR	95%Cl	*p*-value
	No *n* (%)	Yes *n* (%)						
**Individual and house-hold determinants**								
**Sex of child**								
Male	92(50.8)	89(49.2)	1.00	0.76–1.32	0.997			
Female	119(50.9)	115(49.1)	1					
**Child’s age**								
< 24.9	40(46.5)	46(53.5)	1.14	0.89–1.45	0.301			
25-41.9	53(50.0)	53(50.0)	1.06	0.84–1.34	0.618			
≥ 42	118(52.9)	105(47.1)	1					
**Weight-for-Height Z score (WHZ)**								
Wasted (<-2)	7(22.6)	24(77.4)	1.65	1.33–2.05	**< 0.001**	1.44	1.13–1.82	0.003*
Not wasted (≥-2)	204(53.1)	180(46.9)	1			1		
**Weight -for-age Z score (WAZ)**								
Underweight (<-2)	69(38.8)	109(61.2)	1.53	1.26–1.86	**< 0.001**	**1.37**	1.11–1.70	0.004*
Not underweight (≥-2)	142(59.9)	95(40.1)	1					
**Height-for-age Z score (HAZ)**								
Stunted (<-2)	147(47.0)	166(53.0)	1					
Not stunted (≥-2)	64(62.8)	38(37.3)	0.70	0.53–0.92	**0.011**	0.88	0.67–1.16	0.359
**Birth weight**								
< 2.5	26(51.0)	25(49.0)	0.99	0.66–1.51	0.988			
≥ 2.5	185(50.8)	179(49.18)	1					
**Birth order**								
1–3	164(52.2)	150(47.8)	1			1		
4–5	44(50.0)	44(50.0)	1.05	0.75–1.46	0.790	1.16	0.90–1.49	0.263
6 and above	3(23.1)	10(76.9)	1.61	0.85–3.05	**0.145**	**2.00**	1.22–3.29	0.006*
**Mother’s education level**								
None	119(51.1)	114(48.9)	1					
Primary	82(48.0)	89(52.1)	1.06	0.81–1.40	0.662	1.03	0.85 − 1.24	0.798
Secondary	10(90.9)	1(9.1)	0.19	0.03–1.33	**0.094**	**0.19**	0.04 − 0.98	0.048*
**Mother’s age**								
< 25	112(50.0)	112(50.0)	1					
26–35	94(55.6)	75(44.4)	0.89	0.66–1.18	0.424	0.85	0.67 − 1.06	0.155
Above 36	5(22.7)	17(77.3)	1.55	0.93–2.57	**0.094**	1.16	0.82 − 1.63	0.411
**Mother’s Hb level (g/dL); Median (IQR) = 11(10**,**12)**								
< 11	71(49.0)	74(51.0)	1.059	0.80–1.41	0.689			
≥ 11	140(51.9)	130(48.2)	1					
**Number of children**								
1–2	86(49.4)	88(50.6)	1					
3–5	87(53.1)	77(47.0)	0.93	0.68–1.26	0.634			
> 5	38(49.4)	39(50.7)	1.00	0.69–1.46	0.994			
**Average household monthly income**								
< 50,000	113(46.5)	130(53.5)	1					
50,000–100,000	98(57.0)	74(43.0)	0.80	0.60–1.07	**0.135**	0.91	0.75 − 1.12	0.395
**Household size**								
1–3	36(51.4)	34(48.6)	1.05	0.71–1.55	0.802			
4–5 > 5	54(45.0) 121(53.8)	66(55.0) 104(46.2)	1.19 1	0.87–1.62	0.269			
**Mean (SD) ANC visits = 5.4 (± 1.4)**			1.05	0.98–1.13	**0.157**	1.06	0.99 − 1.13	0.092
**Family uses a latrine**								
No	66(50.0)	66(50.0)	1.03	0.76–1.37	0.867			
Yes	145 (51.2)	138(48.8)	1					
**Handwashing facility available**								
No	124(48.4)	132(51.6)	1					
Yes	87(54.7)	72(45.3)	0.88	0.66–1.17	0.375			
**Solid waste disposal area**								
No	66(50.8)	64(49.2)	1.00	0 0.75 − 1.35	0.988			
Yes	145(50.9)	140(49.1)	1					
**Source of drinking water**								
Borehole	66(55.0)	54(45.0)	1					
Protected well	60(53.6)	52(46.4)	1.03	0.71–1.51	0.872			
Tap water	74(48.1)	80(52.0)	1.15	0.82–1.63	0.415			
Rain water	11(37.9)	18(62.1)	1.38	0.81–2.35	0.237			
**Other patient and nutritional-related determinants**								
**History of sickle cell disease**								
No	208(50.7)	202(49.3)	1					
Yes	3(60.0)	2(40.0)	0.811	0.20–3.27	0.769			
**Other comorbidities**								
None	97(49.5)	99(50.5)	1					
Fever	21(61.8)	13(38.2)	0.76	0.42–1.35	0.345			
Malaria	67(49.3)	69(50.7)	1.00	0.74–1.37	0.977			
Diarrhea	22(64.7)	12(35.3)	0.70	0.38–1.27	0.241			
Acute respiratory infections	4(26.7)	11(73.3)	1.45	0.78–2.71	0.241			
**Duration of exclusive breast feeding**								
< 3 months	23(59.0)	16(41.0)	0.79	0.47–1.33	0.366			
3-5months	88(52.7)	79(47.3)	0.91	0.68–1.21	0.509			
6 months	100(47.9)	109(52.2)	1					
*Feeding practices*								
**Cereals**								
No	25(49.0)	26(51.0)	1.04	0.69 − 1.57	0.843			
Yes	186(51.1)	178(48.9)	1					
**White roots and tubers**								
No	57(53.8)	49(46.2)	0.92	0.67–1.27	0.618			
Yes	154(49.8)	155(50.2)						
**Dark green leafy vegetables**								
No	57(46.0)	67(54.0)	1.15	0.86 − 1.54	0.355			
Yes	154(52.9)	137(47.1)	1					
**Vitamin A**								
No	145(51.2)	138(48.8)	1					
Yes	66(50.0)	66(50.0)	1.03	0.76–1.37	0.867			
**Organic meat**								
No	170(50.6)	166(49.4)	1					
Yes	41(51.9)	38(48.1)	0.97	0.68–1.38	0.882			
**Eggs**								
No	168(52.5)	152(47.5)	1					
Yes	43(45.3)	52(54.7)	1.15	0.84–1.58	0.377			
**Fish**								
No	143(46.9)	162(53.1)	1			1		
Yes	68(61.8)	42(38.2)	0.72	0.51–1.01	**0.057**	0.75	0.57–0.99	0.039*
**Legumes**								
No	80(45.7)	95(54.3)	1					
Yes	131(54.6)	109(45.4)	1.20	0.91–1.57	0.204			
**Milk and milk products**								
No	167(47.9)	182(52.2)	1					
Yes	44(66.7)	22(33.3)	0.64	0.41–0.99	**0.047**	0.71	0.48 − 1.05	0.085
**Oils and fats**								
No	146(46.2)	170(53.8)	1			1		
Yes	65(65.7)	34(34.3)	0.64	0.44–0.92	**0.017**	0.70	0.51–0.97	0.029*
**Sweets (such as sugar**,** honey)**								
No	143(49.1)	148(50.9)	1					
Yes	68(54.8)	56(45.2)	0.89	0.65–1.21	0.449			
**Spices and beverages**								
No	59(48.8)	62(51.2)	1.06	0.79–1.43	0.698			
Yes	152(51.7)	142(48.3)	1					
**Drinking boiled or treated water**								
No	162(48.1)	175(51.9)	0.72	0.48–1.06	**0.096**	0.76	0.56 − 1.03	0.075
Yes	49(62.8)	29(37.2)	1					
**HDD score; {Mean (SD) = 5.3(± 2.3)}**			0.95	0.91–0.99	**0.025**	1.04	0.98 − 1.10	0.201

* Refers to significant p-value; cPR- Crude prevalence ratio; aPR-Adjusted prevalence ratio; CI- Confidence interval; HDD score- Household dietary diversity score

## Discussion

Anaemia continues to be a major health problem among children under five years old in Uganda [20]. However, its magnitude among populations in refugee settlements is minimally documented. In the present study, the prevalence and contributors to anaemia among children aged 6 to 59 months in a Kyangwali refugee settlement, Uganda was determined.

This study found 49.1% of the children to be anaemic. The high burden of anaemia found in this study is also reported in other refugee settlements of Uganda that rely considerably on GFA as a source of food and other livelihood needs [29]. In some of the refugee communities, prevalence rates have previously reached up to 79% in Lobule, 74% in Bidibidi and 63% in Palabek [29]. Noticeably, Kyangwali refugee settlement receives only 0.2% of the GFA which is insufficient to sustain the dietary requirements within the refugee community [29]. Yet, similar to other rural refugee settlements, Kyangwali faces limitations in accessing agricultural land, knowledge, and tools necessary for diverse crop production and selling to improve livelihoods [39, 40]. Many of the refugee communities rely on allocated or rental communal land, which may not always available or sustainable [39]. Agricultural productivity is additionally hampered by other calamities including the effects of climate change, crop diseases/ parasites and low prices of produce [41]. Moreover, the reduction in the food rations to refugees (from 100 to 60% of the recommended daily food requirement of 2100Kcal) as a result of the COVID-19 pandemic [29] could have only worsened the situation and may add to the current burden of anaemia in this population. The lack of adequate and sustainable food supply to Kyangwali and related refugee settlements means that the nutritional needs of children cannot be met, resulting in high rates of malnutrition and anaemia. Relatedly, high anaemia prevalence rates of up to 58% were described in children below five years living in refugee communities [42].

The prevalence of anaemia reported in this study also compares to that found in non-refugee populations. A recent nationally representative survey that used the 2016 Uganda Demographic and Health Surveys (UDHS) data showed variation in anaemia patterns with some communities such as Acholi, Teso, Busoga, West Nile, Lango and Karamoja sub-regions that are described as hot spots for childhood anaemia [20]. Other studies carried out in Namutumba district, Eastern Uganda and in Southwest Uganda also found prevalence rates of 59% and 42% respectively [15, 19], suggesting that the burden of anaemia is equally profound amongst non-refugee communities. Therefore, the planning of interventions for reducing childhood anaemia should consider all the other burdened communities.

In this study, the prevalence of anaemia was significantly high among wasted and underweight children. This prevalence also reflects upon the feeding practices of this population. The fact that underfeeding introduces a scope of macro and micro nutrient deficiencies, malnourished children are more likely to be predisposed to anaemia and other deficiency manifestations. This is in line with other studies that suggest a positive relationship between malnutrition and anaemia [17, 19, 21, 22], thus emphasizing the need for screening of children for anaemia at all nutritional clinics and treating anaemia along with other micronutrient deficiencies. Moreover, another study found the risk of anaemia to be higher among participants that reported lower intakes of energy, protein, folate, vitamin B12, iron, vitamin C and red meat [43]. While no significant relationship between anaemia occurrence and the household dietary diversity score was found, this study shows that children who were fed on fish and a source of fat or oils had significantly lower anaemia prevalence. This may be attributed to the wide range of nutrients found in animal-source foods such as fish. Particularly, small dried fish is a rich source of iron, zinc, calcium, folate, vitamin B12, vitamin A and proteins [44, 45] needed for blood formation and proper health. Thus, addressing barriers to the consumption of such nutrient rich foods in refugee communities like Kyangwali may in addition help alleviate the anaemia burden in these communities.

Additionally, anaemia prevalence was associated with the child’s birth order and maternal education. Children of higher birth order may be more susceptible to anaemia due to potential depletion of nutrients such as iron, folate and vitamin B12 in the mother as she ages [43] and with an increasing number of births [46]. This is also in line with other studies that found a relationship between higher birth order and anaemia [17, 21]. These findings emphasize need of addressing nutritional needs for aging and multiparous mothers so as to prevent anaemia risk in the children delivered by these mothers. The prevalence of anaemia decreased with increase in mother’s level of education, as reported in other studies [4, 15, 17, 20, 21]. In this study, more than half of the mothers did not have any formal education, highlighting the need for programs that promote education within refugee communities. Also notably, the coverage of deworming practices in Kikuube district at 58.2% is inadequate [9] and may further contribute to the burden of anaemia among this population.

Anaemia and malaria are also strongly related and can have severe effects in children. Affected children may be subjected to stunted growth, impaired cognitive and motor development and increased susceptibility to infections [1, 12]. Although malaria was not significantly associated with anaemia, about one third of the study children presented with malaria and half of these had anaemia. Previous studies among Kenyan and Malawian populations reported an increased risk of recurrent severe anaemia and post-discharge mortality among children admitted with anaemia and malaria parasitaemia [13, 14]. Another study done in Kenya and Uganda found a reduced risk of severe anaemia among children administered with malaria chemoprevention after discharge [47]. These findings raise need for creation of awareness on the post-discharge risks for children admitted with severe anaemia and advocating for uptake of available interventions proven to reduce malaria and anaemia in children.

### Study limitations

There were some limitations in this study. Except for patient data, information on other determinants of childhood anaemia was obtained by self-report interviews. Data based on self-reporting are limited by information or response bias. This could have influenced the outcome of the multivariate analysis since some of the known determinants of anaemia such as the HDD score unexpectedly showed no association with anaemia prevalence. Additionally, the cross-sectional nature of this study restricted our ability to assess temporal or causal relationships.

## Conclusions

This study showed that 49% of the children aged 6–59 months in Kyangwali refugee settlement were anaemic. The most significant contributors to anaemia in this population were malnutrition, maternal education, feeding practices and birth order. Results from this study emphasize the need for screening of children for anaemia at all nutritional clinics, promotion of education, addressing barriers to sustainable food supply and accessibility of nutrient-dense foods, treating anaemia in children alongside other micronutrient deficiencies and addressing the nutritional needs of multiparous mothers. In addition, the study findings raise need for creating awareness on the post-discharge risks for children admitted with anaemia, as well as improving the uptake of available interventions for reducing malaria and anaemia in children. This may help alleviate the burden of anaemia among children in this population.

## Data Availability

No datasets were generated or analysed during the current study.

## Abbreviations

WHO: World Health Organisation
IFA: Iron-Folic Acid
UNHCR: United Nations High Commission for Refugees
WFP: World Food Programme
GFA: General Food Assistance
HC: Health Centre
IPC: Integrated food security Phase Classification
YCC: Young Child Clinic
Hb: Haemoglobin
Kg: Kilogram
cm: Centimetre
WAZ: Weight-for-Age Z score
HAZ: Height-for-Age Z score
WHZ: Weight-for-Height Z-score
SD: Standard Deviation
HDD score: Household Dietary Diversity score
ANC: Antenatal Care
IQR: Interquartile Range
PR: Prevalence Ratio
CI: Confidence interval
DHS: Demographic Health Survey
CIDI: Community Integrated Development Initiative
